# Experimentally Induced Cerebral Cystic Echinococcosis in Rats: A Suitable Animal Model for Cerebral Echinococcosis

**Published:** 2020

**Authors:** Mohammad Hossein RADFAR, Soheila FOTOOHI, Shahrzad AZIZI, Reza KHEIRANDISH

**Affiliations:** Department of Pathobiology, School of Veterinary Medicine, Shahid Bahonar University of Kerman, Kerman, Iran

**Keywords:** *Echinococcus granulosus*, Experimental, Hydatid cyst, Cerebral

## Abstract

**Background:**

*Echinococcus granulosus* is a worldwide zoonotic cestode that lives mainly in the intestine of dog as definitive host. Its larval stage infects intermediate hosts and forms hydatid cysts mainly in the liver and lungs tissues and less other organs such as brain, eye and bone. In the experimental models, inoculation of protoscoleces into the peritoneum, thoracic cavity, subcutaneous and cerebrum produces hydatid cysts. Experimental echinococcosis in the animal models provides a good opportunity for study of the parasite-host relationship, different transmission ways of infection in the intermediate hosts and effect of new drugs.

**Methods:**

The present study was conducted in the Veterinary School, Shahid Bahonar University of Kerman, Kerman, Iran in 2018. In this study, cerebral hydatidosis was investigated in 6 female Wistar rats weighing (200±20 gr). For this purpose, protoscoleces were collected from hydatid cysts of infected sheep liver. Overall, 300 protoscolices were injected directly in the lateral ventricle by an insulin syringe through the implanted cannula.

**Results:**

After 4 months of inoculation, multiple thin-walled, transparent hydatid cysts were observed in the rat skull. All cysts were infertile. The cysts were localized prominently on the cerebral cortex and lesser in the parenchyma and ventricles. The cyst walls consisted of three layers consist of the outer layer (fibrous capsule), two parasitic layers and the endocyst layer (germinal layer). The cyst was surrounded by the inflammatory cells consist of lymphocytes, plasma cells and macrophages.

**Conclusion:**

To the best of our knowledge, this research is the first experimental cerebral hydatidosis arisen from larval stage of *Echinococcus granulosus* in the animal model.

## Introduction

E*chinococcus granulosus* is a cestode with worldwide distribution. Adult form of this helminth lives in the intestine of definitive hosts such as dogs and other canids. Its larval stage (metacestode) infects the intermediate hosts including livestock and humans and causes cystic echinococcosis (CE) or hydatid disease (1). Ingested *Echinococcus* eggs containing oncosphere develops into primary metacestodes (or hydatid cysts) in the intermediate hosts. Secondary CE occurs when hydatid cysts rupture in the intermediate host and released protoscolices form new cysts. Hydatid disease threats public health and has economic and social importance (2). Moreover, about 2%±4% mortality rate is reported for hydatidosis that may be increased if medical treatment is not effective.

Numerous studies have been carried out in the forms of in vivo and in vitro on the *E. granulosus*, and some experimental models have been done to make secondary hydatid cysts. In the experimental model of secondary CE, inoculation of protoscolices into the peritoneum, thoracic cavity, subcutaneous and cerebrum can produce hydatid cysts. Experimental echinococcosis in the animal models provides a good opportunity for study of the parasite-host relationship, immunological analytic, different transmission ways of infection in the intermediate hosts and effect of new drugs (3). Many aspects of interaction between the intermediate hosts and *E. granulosus* are not obvious and should be elucidated. Hydatid cysts grow mainly in the liver and lungs and less in other organs such as bone, orbits and brain (2, 4). Intracranial hydatid cysts is associated with or without cyst in other organs of the body (5, 6). Intracranial hydatid cysts are commonly solitary (7). Multiple intracranial cysts are rare (8).

In the present study, hydatid cysts were established in the brain of rat by intracranial injection of echinococcal larvae.

## Materials and Methods

### Statement of ethics committee

All animals received human care in compliance with the Guide for Care and use of Laboratory Animals published by the National Institutes of Health (NIH publication No. 85–23, revised 1985). The study was approved by Institutional Animal Care and Use Committee of our veterinary school (Shahid Bahonar University of Kerman, Kerman).

### Experimental animals

The present study was conducted in the Veterinary School, Shahid Bahonar University of Kerman, Kerman, Iran in 2018. Six female Wistar rats weighing (200±20 gr) were provided from Kerman Neurosciences Research Center. Animals were housed in the standard condition with temperature (21±2 °C) and light-cycle (12 h light-dark cycles). They had free access to standard food and freshwater.

### Protoscolices collection

Protoscolices of *E. granulosus* strain G1 were aseptically collected from the hepatic hydatid cysts of naturally infected sheep in an abattoir located in Kerman province, Iran. Cyst fluid was aspirated with a 50 mL syringe. Adhered protoscolices to cyst wall were separated from the endocyst with repeated aspiration and emptying of fluid cyst. Then, the wall cyst was opened and the internal surface washed by physiological saline. The whole fluids including the physiological saline and cyst fluid were completely collected and kept. This sample was centrifuged at 3000 r/min for 5 min and the supernatant was removed. The sediment was washed with PBS containing gentamicin three times for removing fragments of vesicles, necrotic tissues and blood components until a pure protoscolices obtained. Viability of protoscolices was determined by 0.1% eosin staining under an optical microscope. Protoscolices were diluted with the normal saline to reach a concentration of 300 protoscolices/7μl.

### Intracranial injection of protoscolices by cannula

**Fig. 1:**
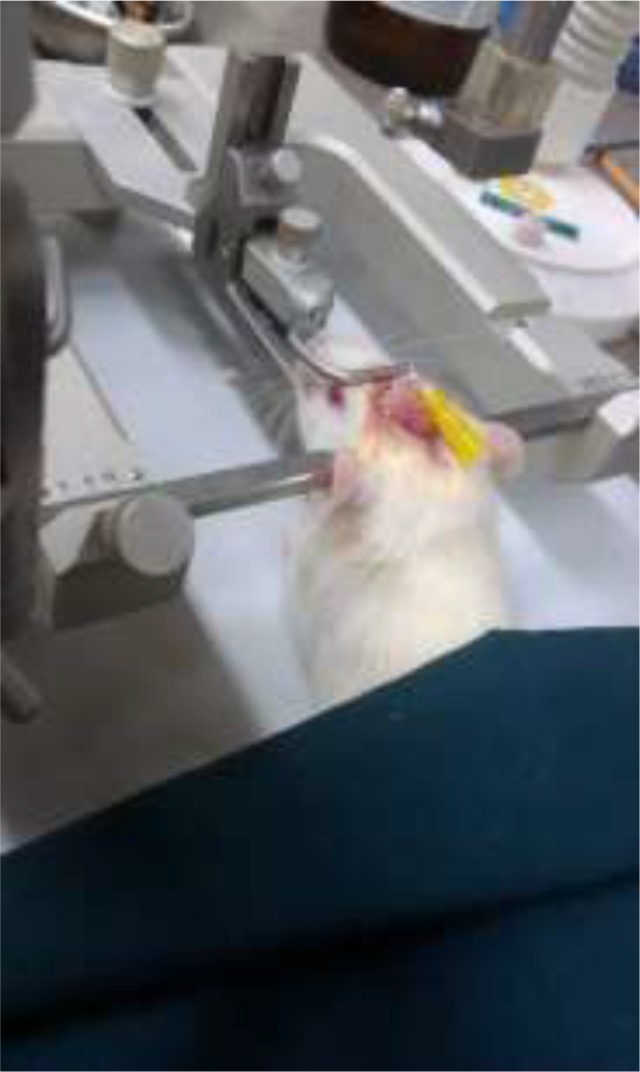
Inserted cannula directly at lateral ventricle (−0.92 mm posterior, +1.2 mm lateral to midline and 3.3 mm ventral)

**Fig. 2:**
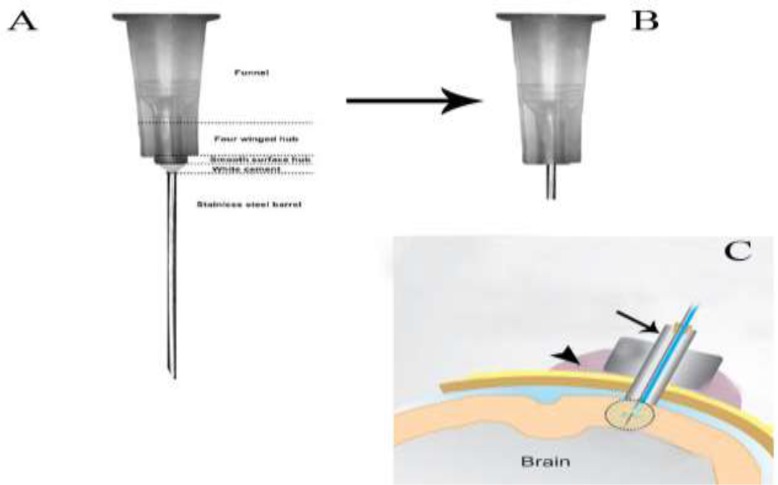
The guide cannula is graphically represented. A) a 22 gauge needle, B) remove the top funnel portion, C) the guide cannula (arrow) is implanted in the skull and hardened with dental cement (arrowhead)

Six rats were anesthetized with intramuscular injection of xylazine 2% (4 mg/kg) and ketamine 10% (60 mg/kg) (Alfasan International Group of Companies, Holland). After sterilization, skull skin was opened. With a stereotaxic apparatus (Stoelting Co., USA) and use of a stereotaxic atlas, the accurate anatomical location of lateral ventricles was determined in according to the Paxinos and Watson rat brain atlas. A 22 gauge guide cannula was cut its stainless steel barrel and remained 1 cm of its length. Then, guide cannula was implanted unilaterally in the lateral ventricle under aseptic condition (Fig. 1). Guide cannula became stable to the skull with the stainless steel screws and was tightened rapidly with polymerizing dental cement to the surface of the skull (Fig. 2). After the hardening of the cement, the animal was removed from stereotaxic frame. 300 protoscolices were injected intracranially by an insulin syringe with a blunt-tipped 27-gauge needle through the cannula (9). After that, rats were housed individually.

### Development of echinococcus cysts in the rats

After 4 months of inoculation, the rats were killed by cervical dislocation. No mortality was observed during this course. The hydatid cysts were observed in 5 out of 6 inoculated rats. Some samples of brain containing echinococcal cysts were taken and fixed in the neutral buffered formalin 10%. After fixation, they passed other stages in the autotechnicon tissue processor according to the standard procedure. About 5 μm thicknesses were provided from the paraffin-embedded tissues and stained with HE staining for histopathologic study.

## Results

### Pathologic findings

**Fig. 3:**
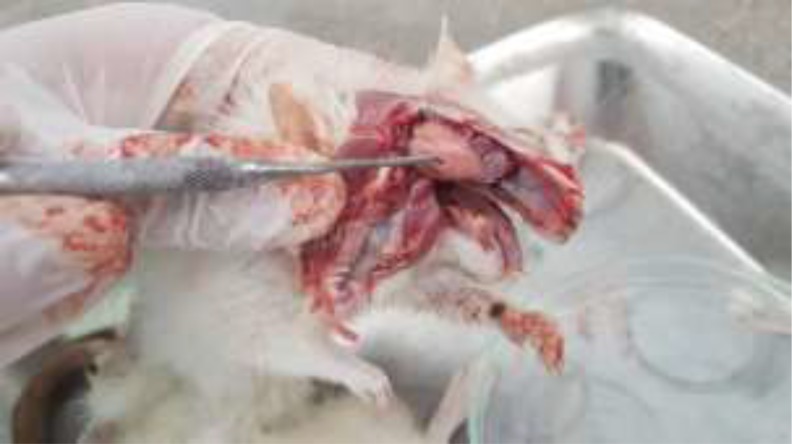
Experimental echinococcosis in rat. Multiple transparent thin-walled, hydatid cysts are created in the skull

**Fig. 4:**
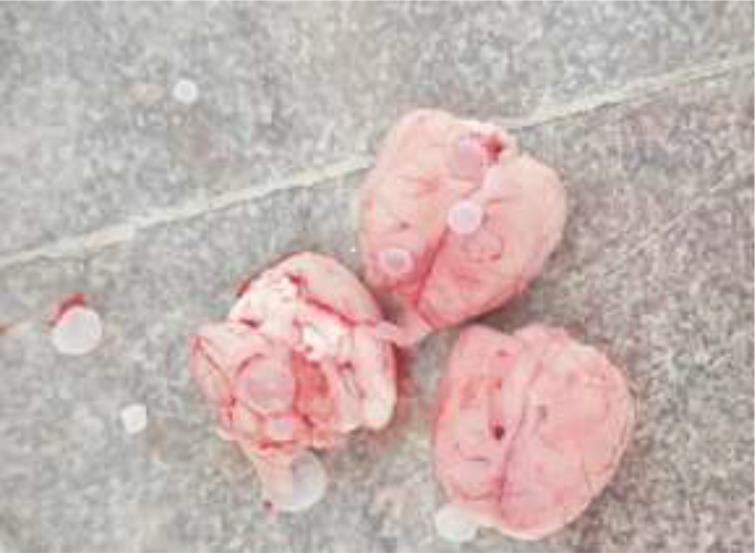
Rat infected with *Echinococcus granulosus*. Hydatid cysts in different size are visible on the parietal lobes and also ventral surface of brain

**Fig. 5:**
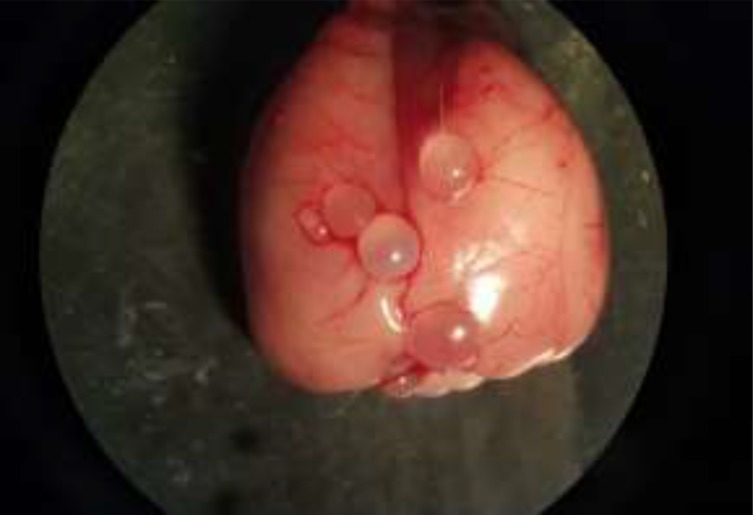
Fluid-filled, spherical, unilocular cysts related to *E. granulosus* in rat

Macroscopic examination of the rat brains revealed multiple transparent thin-walled, cysts. The cysts were round in shape and fluid existed in their cavities. The size of cerebral cysts varied from 3–7 mm (Fig. 3–5). All cysts were infertile. They adhered to the surrounding tissues.

**Fig. 6:**
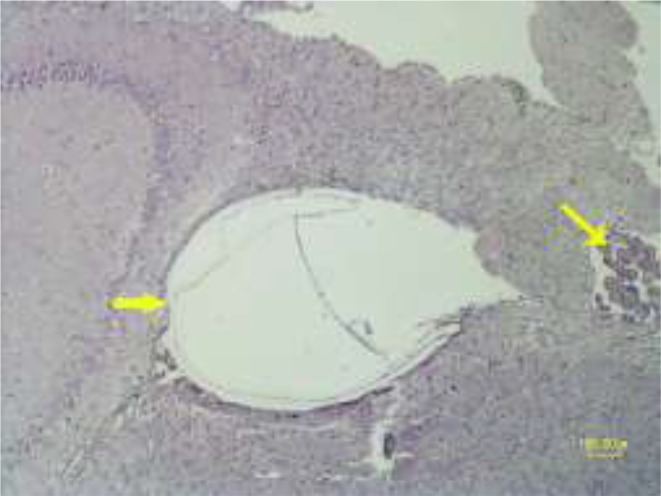
Histopathology of cerebral echinococcosis. Formation of hydatid cyst in the lateral ventricle of rat (thick arrow). Choroid plexus is obvious in the ventricle (thin arrow) (HE, Bar=100μm)

**Fig. 7:**
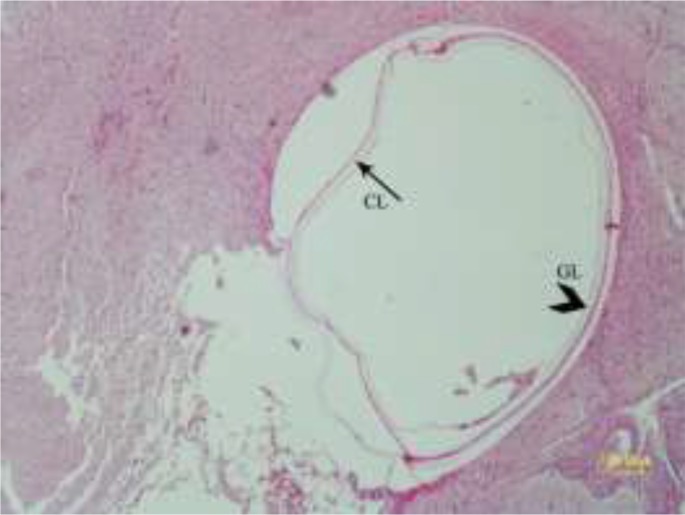
Experimental hydatidosis. Cerebral cyst wall is formed from an acellular layer (cuticle layer: Cl) as well as a germinal layer (Gl) (HE, Bar=100μm)

**Fig. 8:**
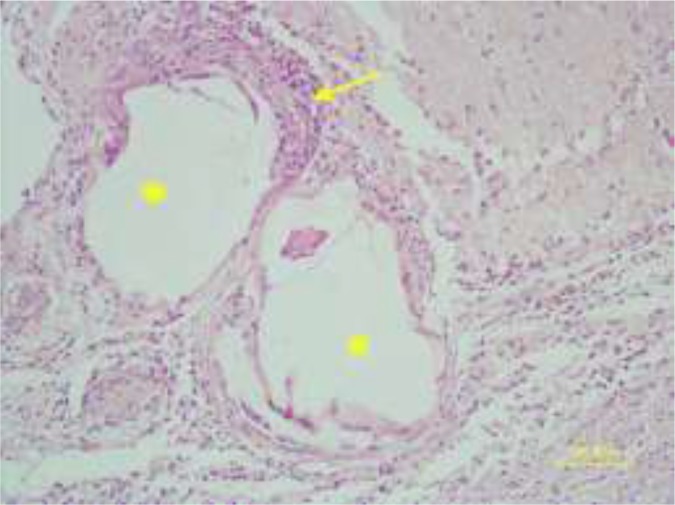
Mononuclear inflammatory cells reaction (thin arrow) around the hydatid cysts (asterisks) in rat brain (HE, Bar=100μm)

**Fig. 9:**
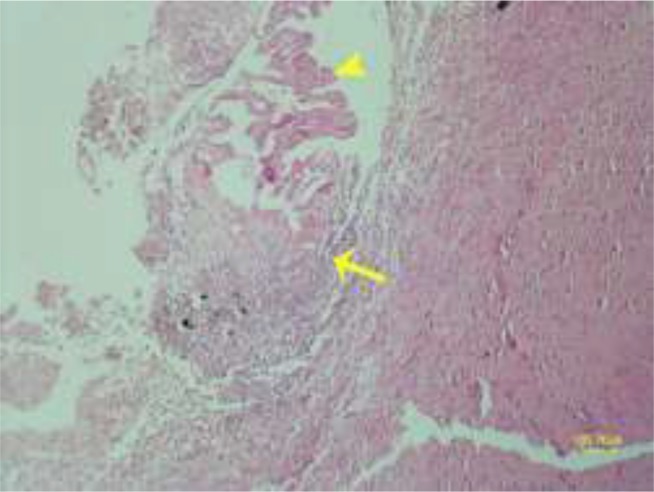
Choroiditis is caused due to presence of hydatid cysts in the ventricle. Choroid plexus (arrowhead) and inflammatory cells (arrow) are shown in the figure (HE, Bar=100μm)

In the histopathologic examination, they were observed more on the cerebral cortex and lesser in the brain parenchyma and within the lateral ventricles (Fig. 6). The cyst walls consisted of three layers consist of the outer layer or fibrous capsule (Fc) that is a defense mechanism of host tissue. Within this fibrous capsule, there were two parasitic layers including an acellular layer (cuticle layer: Cl) with component of mixed mucopolysaccharide and proteins and other, the endocyst layer named germinal layer (Gl) (Fig. 7). No protoscoleces were found in the Gl layer of cysts. The fibrous tissue of echinococcal cyst was surrounded by the inflammatory cells consist of lymphocytes, plasma cells and macrophages infiltrated around the cysts (Fig. 8). Choroiditis due to presence of cysts in the ventricles was observed (Fig. 9). According to microscopic characteristics, the cysts were confirmed as hydatid cyst.

## Discussion

Echinococcosis is a chronic zoonotic disease that occurs in the result of infection by the larval stages of taeniid cestodes (10). *E. multi-locularis* and *E. granulosus* are important pathogenic species for human that lead to alveolar and cystic echinococcosis, respectively. The most common affected organ is liver (77%) and after that lungs (43%) (11). Hydatid disease rarely involves CNS and only 1%–2% of all hydatid infections are related to the brain (12, 13). The cysts are 2–3 times more common in the brain of children and young adults (50%–75%) (13, 14). Humans may be infected due to ingestion of contaminated food by dog feces, or direct contact with dogs. The eggs hatch in the stomach and small intestine. The released larvae penetrate into the vessels of gut wall and then enter the liver where the hydatid cysts form. Rarely, the larvae may bypass the hepatic sinusoids into the circulatory system and reach the lungs and occasionally the brain (15).

Investigation of *Echinococcus* spp. in animal models can be useful to determine the pathogenesis of this parasite for finding new control programs in future, understanding new treatments, surgical approaches and producing vaccine. Experimental echinococcosis was administrated by various methods such as gavage echinococcal eggs or intraperitoneal injection of protoscolices in rodents (16, 17).

In the present study, cerebral hydatidosis was created in rats. Typical hydatid cysts with cuticle (Cl) and germinal layer (Gl) were formed in CNS by intracranial injection of protoscolices obtained from ovine hydatid cysts. Mononuclear inflammatory cells surrounded the outer layer of cysts.

The echinococcal cyst (metacestode) is filled with fluid, spherical in shape, unilocular and consists of an inner germinal layer of cells supported by an acidophilic-staining, acellular and laminated membrane A host-produced reaction of granulomatous inflammation surrounds each cyst. Small vesicles bud from the germinal layer and a number of protoscolices develop by asexual division (18).

There are few studies on experimental cerebral echinococcosis (19). Secondary alveolar echinococcosis (AE) was investigated in the brains of rats by using magnetic resonance imaging (MRI) and immunoblot (western blot) techniques (9). They injected 10% homogenate of echinococcal larval tissues intracranially in the female rats. Two weeks after injection, T2-weighted MR images revealed a hyperintense region in the cerebral cortex. After three weeks, the cysts were observed on the basis of histologic examination. Two epitopes antibodies (Em18 and Em16) of *E. multilocularis* were detected for AE diagnosis by western blot analysis of blood serum after nine weeks of injection. The MRI method was suitable for early detection of secondary cerebral AE. The exact location, size and number of hydatid cysts in the brain can be identified with CT scan. However, MR is widely used as a diagnostic method and is superior to CT scan because shows some details not determined by CT (20).

Based on phylogenetic studies, *E. granulosus* s. l. consists of several distinct genotypes including *E. granulosus* sensu stricto (genotype G1– G3), *E. equinus* (G4) *E. ortleppi* (G5) and *E. canadensis* (G6–G10) (21). In the present study, protoscolices *of Echinococcus granulosus* (G6) were used for experimental hydatidosis in rat.

Sadjjadi et al. investigated molecular characteristics of cerebral *Echinococcus* cysts in 10 specimens of cerebral *Echinococcus* cysts. BioEdit and BLAST software analysis indicated that sequenced isolates belong to the *E. granulosus* (G6) genotype. All of brain cysts belonged to the G6 genotype and all of the liver cysts to G1. G6 has a higher affinity for the human brain than G1 (22).

In our study, the hydatid cysts had been localized on the cerebral cortex and less intraparenchyma and in the ventricles. Similarly, intracranial hydatid cysts manifest majorly on the parietal lobes in humans (23, 24) and sometimes in other sites of CNS such as pons, cerebellum and ventricles (7).

On the base of our knowledge, no report of hydatid cysts is present in animals and cerebral hydatidosis is a rare manifestation in humans. The most human cysts are isolated from supratentorial and intraparenchymal in the middle cerebral artery territory (25), and a few of them may involve the ventricles (26).

The hydatid cysts in CNS growth slowly between 1.5–10 cm/year and is diagnosed when become large (27) but in a report, large multiple hydatid cysts were diagnosed within 6–12 months after the surgery that suggests a higher growth rate. The clinical signs of cystic echinococcosis are depended on the location and size of the cysts (28). Neurologic dysfunction such as headache, vomiting, epilepsy and dysphasia appear in humans affected by the cerebral echinococcosis (29, 30). Surgery is the choice treatment for large cerebral hydatid cysts with aim of the intact delivery cysts (31). Dowling’s hydrodissection technique is the most useful method for intact removal of cysts (32).

Control of hydatid disease especially in developing countries is important. Educations of rural populations, treatment of infection in the definitive and intermediate hosts and vaccination in some countries are effective in reduction of echinococcosis. In research in Australia and Argentina, 86% of vaccinated sheep were free of hydatid cyst until 1 year after immunization by recombinant vaccine (EG95) (33). In other study, 97%–100% protection against growth of tapeworm and egg production is reported by vaccination of dogs (34). In animal models, vaccinated BALB/c mice with rBCG-EgG1Y162. IgG and IgE against the recombinant protein were detected by ELISA. The rBCG-EgG1Y162 vaccine induced strong and specific cellular and humoral immune responses and can be a new candidate for reducing the risk of human infected by *E. granulosus* (35).

We herein described an experimental method for establishing cyst of *E. granulosus* in the brain. It seems rat is suitable animal model for induction of secondary hydatid cysts in brain. Wistar rat is highly susceptible to the secondary infection in contrast to *E. multilocularis* infection through orally inoculation of echinococcus egg (36). The results of current study are preliminary and can be helpful for more research in future. Further investigations are needed to elucidate the pathogenesis of hydatid disease, growth rate of cysts, main site and etiology of cysts localization and new control program of this disease.

## Conclusion

To the best of our knowledge, this research is the first experimental cerebral hydatidosis due to larval stage of *E. granulosus* in the animal models.
